# Distribution of *CYP2D6* and *CYP2C19* gene polymorphisms in Han and Uygur populations with breast cancer in Xinjiang, China

**DOI:** 10.1515/biol-2022-0728

**Published:** 2024-04-20

**Authors:** Muzhapaer Abudukeremu, Aisikaer Ayoufu, Adila Tuerhong, Xuelaiti Paizula, Jiang-Hua Ou

**Affiliations:** Department of Breast Cancer Surgery, Affiliated Tumor Hospital, Xinjiang Medical University, No. 789, Suzhou Road, Urumqi, Xinjiang, 830011, China; Department of Breast and Thyroid Surgery, Affiliated Cancer Hospital of Xinjiang Medical University, Urumqi City, 830000, Xinjiang, China

**Keywords:** cytochrome P450 enzymes, tamoxifen, drug metabolism, oestrogen receptor, *CYP2D6*, *CYP2C19*, gene polymorphism

## Abstract

The aim of this study was to investigate the frequency distribution of the cytochrome P450 (*CYP450*) enzymes, *CYP2D6* and *CYP2C19*, and the form of tamoxifen metabolisation in premenopausal patients with breast cancer in the Han and Uygur ethnic groups of Xinjiang to guide rational clinical drug use. A total of 125 Han patients and 121 Uygur patients with premenopausal hormone-receptor-positive breast cancer treated at the Xinjiang Uygur Autonomous Region Cancer Hospital between 1 June 2011 and 1 December 2013 were selected. The common mutation sites in *CYP450* were analysed using TaqMan® minor groove binder technology. Genetic testing was performed to determine other metabolic types of tamoxifen, and the genotypes and metabolic types were compared using a Chi-squared test. Between the Han and Uygur groups, there were significant differences in the frequencies of the *CYP2D6* (*10/*10) and *CYP2C19* (*1/*1) genotypes, with *P-*values of 0.002 and 0.015, respectively. Genotypes of *CYP2D6* (*1/*1), *CYP2D6* (*1/*5), *CYP2D6* (*5/*5), *CYP2D6* (*5/*10) and *CYP2C19* (*3/*3) were expressed in the two patient groups, and the difference was not statistically significant (*P* > 0.05). In the Han patients, the proportions of extensive, intermediate and poor metabolisers of tamoxifen were 72, 24 and 4%, respectively, whereas those in the Uygur patients were 76.9, 17.4 and 5.7%, respectively, with no significant difference (*P* > 0.05). In conclusion, There were partial differences in the *CYP2D6* and *CYP2C19* gene polymorphisms of *CYP450* between the Han and Uygur patients with premenopausal breast cancer, but there was no significant difference between the *CYP2D6* and *CYP2C19* phenotypes. Further research is needed to determine the relationship between the enzyme genetic differences of *CYP450* and the pharmacokinetics and efficacy of tamoxifen. Although there were some differences in genotypes, these did not result in differences in the predicted tamoxifen metabolisation phenotype between the Han and Uygur patients with breast cancer. Therefore, the doses should be adjusted according to the individual genotype data.

## Graphical abstract

**Figure:**
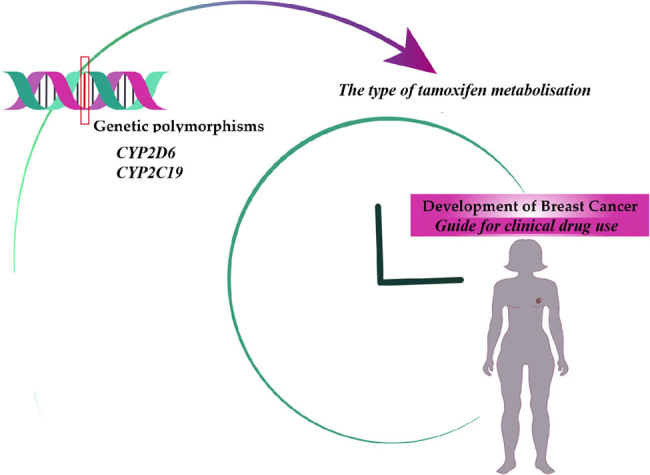

## 1 Introduction

The Chinese population is known for its rich genetic diversity, with significant inter-/intra-ethnic variation in the frequency distribution of germline variants. Understanding this variation is critical for accurately characterising disease risk and developing tailored medical treatments [1]. The results of Wang et al.’s research demonstrated that *ERCC1*/*XPF* genetic polymorphisms in the Uygur population predispose individuals to breast cancer [2]. Cytochrome P450 (*CYP450*) enzymes play an important role in the metabolic process of organisms [3]. Among them, *CYP2D* is located on chromosome 22 and comprises *CYP2D6*, *CYP2D7P* and *CYP2D8P*, which mainly encode and express enzymatically active proteins involved in metabolic processes (*CYP2D7P* and *CYP2D8P* do not code for expressed proteins).

A previous study [4] revealed that poor *CYP2D6* metabolisers are likely to experience an impaired response to tamoxifen, and ultrarapid *CYP2D6* metabolisers are at risk of an exaggerated response with pronounced adverse effects. During drug metabolism, *CYP2D6* not only exhibits stereoselectivity for drugs but also ethnic and individual differences. The main reason for this difference pertains to *CYP2D6* genetic polymorphism. Different gene alleles can cause variations in the number and activity of enzymes, meaning drug metabolism presents individual differences [5]. Polymorphism of the *CYP2D* gene can also affect an individual’s adverse reactions to certain drugs. For example, adverse reactions to certain antidepressants and painkillers are related to the polymorphism of the *CYP2D6* gene. By detecting *CYP2D* gene polymorphisms, whether an individual will experience adverse reactions can be predicted and drug doses can be adjusted or alternative treatment plans selected accordingly. Similarly, by studying *CYP2D6* gene polymorphisms, an individual’s metabolism rate of certain drugs can be predicted and personalised treatment plans subsequently developed [6].

Tamoxifen, an oestrogen-receptor modulator, plays a key role in the adjuvant and palliative treatment of breast cancer. It is clinically significant in reducing the mortality of patients with oestrogen-dependent breast cancer and improving their quality of life [7]. The difference in the expression of drug-metabolising enzymes is a determinant of individual efficacy and side effects, and the genetic difference in *CYP450* enzymes significantly impacts the effect of antitumor drugs [8].

Clinically, approximately 50% of patients with hormone-dependent breast cancer benefit from tamoxifen [9]; this difference in efficacy may relate to ethnic and individual differences in *CYP2D6* genes and *CYP2C19* genotypes [6,7]. Studies have demonstrated that the CYP2D6*10/*10 genotype is associated with the curative effect of tamoxifen in breast cancer [10]. A meta-analysis revealed that the concentration of endoxifen was significantly higher in patients with CYP2D6*10 (the C/C genotype) than in patients with CYP2D6*10 (the CT/TT genotype) [11]. However, studies have identified no correlation between the CYP2D6*10 genotype and the survival rate of patients with breast cancer receiving tamoxifen therapy [12]. It is reported that the deletion of CYP2D6*5 can lead to the loss of the entire *CYP2D6* metabolic function.

Based on their functions, *CYP2D6* enzymes can be divided into four phenotypes: ultrarapid, extensive, intermediate and poor metabolisers [13]. Clinical studies have revealed differences in the efficacy of tamoxifen that may relate to ethnic and individual differences in the *CYP2D6* gene and *CYP2C19* (rs3758581) genotype [6,7]. Moreover, Wei et al. confirmed that *CYP2C19* polymorphisms do not significantly impact tamoxifen metabolism or breast cancer relapse [14].

In summary, studying the polymorphism of the *CYP2D* gene can support personalised drug treatment and help us better understand human genetics and biology.

We hypothesised that the polymorphisms of the *CYP2D6* and *CYP2C19* genes can affect the efficiency of tamoxifen in the treatment of patients with breast cancer. Thus, the genotypes and metabolic types of two different ethnic groups were determined to explore the ethnic differences between *CYP2D6* and *CYP2C19* genetic polymorphisms in *CYP450* to improve the gene pool of various ethnic groups and provide an important basis for personalised medicine.

## 2 Materials and methods

### 2.1 Basic patient information

A total of 125 Han and 121 Uygur premenopausal patients with breast cancer admitted to the Department of Breast Surgery at the Xinjiang Uygur Autonomous Region Cancer Hospital between 1 June 2011 and 1 December 2013 were selected as the research participants. All patients were confirmed by immunohistochemistry as hormone-receptor positive (oestrogen receptor+ and [or] progesterone receptor+), with a pathological diagnosis of invasive breast cancer. The patients’ ages ranged from 31 to 53 years, with a median age of 42. Based on the American Joint Committee on Cancer’s seventh edition of the tumour-node-metastasis *Classification of Malignant Tumours* clinical staging criteria for breast cancer, there were 138 cases of Stage I cancer and 108 cases of Stage II cancer [15].

Table 1 presents the patients’ basic information. All patients had taken or were about to take tamoxifen. The patients’ body mass indexes were within the normal range, and their blood pressure, routine blood and urine tests, liver and kidney functions and electrocardiograms were all normal. There was no statistical difference in the above indicators between the two patient groups, and they were comparable. None of the patients had a history of taking psychiatric drugs or other drugs that can affect the metabolisation of tamoxifen, and all patients were informed that the aim of this investigation is to explore the relationship between *CYP2D6* and *CYP2C19* gene polymorphisms and the form of tamoxifen metabolisation in premenopausal patients with breast cancer in the Han and Uygur ethnic groups of Xinjiang before the test.

**Table 1:** Basic information of patients

Ethnic	Han ethnic	Uygur ethnic
Patients number	125	121
Median age, years	40	43
Breast cancer Stage I	72	66
Breast cancer Stage II	53	55
Lymph node positive, %	38	42
Tumor size >2 cm, %	34	39
BMI, median (IQR), kg/m^2^	26 (22–29)	27 (22–29)
Postmenopausal, %	100	100
**Centrally assessed tumour features**		
ER absent, %	2	1
ER absent, %	5	7

BMI: body mass index; IQR: the interquartile range; ER: estrogen.

This study was approved by the Medical Ethics Committee of the Xinjiang Uygur Autonomous Region Cancer Hospital, and all the participants provided signed informed consent.


**Informed consent:** Informed consent has been obtained from all individuals included in this study.
**Ethical approval:** The research related to human use has been complied with all the relevant national regulations, institutional policies and in accordance with the tenets of the Helsinki Declaration, and has been approved by the Ethics Committee of Affiliated Tumor Hospital, Xinjiang Medical University.

### 2.2 Reagents and materials

Tiangen Biochemical Technology (Beijing, China) provided a blood genomic deoxyribonucleic acid (DNA) extraction kit. A TaqMan^®^ original magnification probe (TaqMan^®^ Single Nucleotide Polymorphism Genotyping Assays, Thermo Fisher Scientific, Waltham, MA, USA; 4362691) and a TaqMan^®^ Genotyping Master Mix were purchased from Applied Biosystems. A GeneAmp™ polymerase chain reaction system (PCR System 9700; Applied Biosystems, Foster City, CA, USA) and a 7900HT Fast Real-Time PCR System with supporting software (version 2.3; Applied Biosystems) from Applied Biosystems were also used to complete the experiment.

## 3 Experimental method

Blood genomic DNA extraction was performed in accordance with the instructions for the Tiangen blood genomic DNA extraction kit, and the purity of the samples was qualified using PCR. All amplification reaction systems were 50 μL and included the following: Taq DNA polymerase (1.25 U); magnesium chloride (100 μmol/L); deoxyadenosine triphosphate, deoxycytidine triphosphate, deoxyguanosine triphosphate and deoxythymidine triphosphate (10 μmol/L); and genomic DNA (4 μL). The upstream and downstream primers were 20 μmol/L each. Pre-denaturation was conducted at 94°C for 5 min followed by denaturation at 94°C for 45 s (the annealing temperatures were 55 and 58°C for CYP2C19*2 and CYP2C19*3, respectively, and 60°C for CYP2D6*10). There was an extension at 72°C for 1 min, with 35 cycles, followed by a final extension at 72°C for 7 min. The test was repeated three times.

Following this, a probe was added to the reaction system. The following reagents were used: Master Mix (3.1 μL), 10× probe (0.16 μL), double-distilled water (1.94 μL) and the DNA sample to be tested (1.2 μL) (if it was a standard, 1 μL was added), up to a total of 6.4 μL. The liquid was added to the sample, and the sample was then mixed in a vortex for 5 s. The test was repeated five times.

Finally, the corresponding gene loci were read, and the metabolic type was determined using the 7900HT system software (version 2.3). The genotype of *CYP2C19* and *CYP2D6* was also confirmed using a gene microarray and was divided into three metabolic groups: weak, intermediate and strong. The metabolic phenotype of *CYP2D6* was divided into four types: ultrarapid metabolisers, extensive metabolisers, intermediate metabolisers and poor metabolisers [10]. The sample size was determined using a sample calculation formula known as Andrew Fisher’s formula. The confidence level was 80%, and the *Z* score was 1.44.

### 3.1 Statistical methods

The experimental data were analysed using SPSS 17.0 software (International Business Machine, Armonk, NY, USA) and were described by rate or composition ratio (%). The Chi-squared (*χ* ^2^) test and Bonferroni correction were used to compare the differences in the distribution of genes and metabolic types between the Han and Uygur patients, with a test level set at *α* = 0.05.

## 4 Results

### 4.1 Genotype frequencies of *CYP2D6* and *CYP2C19*

Table 2 summarises the genotype frequencies of *CYP2D6* and *CYP2C19* in the Han and Uygur premenopausal patients with hormone-receptor-positive breast cancer. It was clear that the differences between the two groups of patients in the genotype frequencies of *CYP2D6* (*10/*10) and *CYP2C19* (*1/*1) were statistically significant. Furthermore, the frequency distribution of genotypes at each locus was consistent with the Hardy–Weinberg equilibrium test (*P* > 0.05); therefore, it can be assumed that the control samples selected for this study were representative of the same population (Table 2). Table 3 presents the allele frequencies of *CYP2D6* and *CYP2C19*.

**Table 2:** Frequency of CYP2D6 and CYP2C19 genotyping in the two patient groups

		Han ethnic group (*n* = 125)		Uygur ethnic group (*n* = 121)		*χ* ^2^ value	*P* value
Gene		Actual frequency in patients	Observed frequency	Actual frequency in patients	Observed frequency		
**CYP2D6**						1.758	0.415
	*1/*1	24	0.19	30	0.25	1.123	0.289
	*1/*5	0	0	0	0	—	—
	*5/*5	0	0	0	0	—	—
	*1/*10	60	0.48	72	0.6	3.272	0.07
	*10/*10	41	0.33	19	0.16	9.746	0.002
	*5/*10	0	0	0	0	—	—
**CYP2C19**						0.001	0.999
	*1/*1	42	0.34	24	0.2	5.935	0.015
	*1/*2	48	0.38	60	0.5	3.124	0.077
	*2/*2	18	0.14	27	0.22	2.576	0.108
	*1/*3	6	0.05	6	0.05	0.003	0.954
	*3/*3	0	0	0	0	—	—
	*2/*3	11	0.09	4	0.03	3.241	0.072

**Table 3:** Allele frequencies of CYP2D6 and CYP2C19 in the two groups

Gene	Allelic genes	Allele frequency		*P* value
		Han ethnic group (*n* = 125)	Uygur ethnic group (*n* = 121)	
CYP2D6	*1	0.432	0.545	0.009
	*5	0.000	0.000	—
	*10	0.568	0.455	0.012
CYP2C19	*1	0.552	0.471	0.073
	*2	0.380	0.488	0.016
	*3	0.068	0.041	0.194

### 4.2 Comparison of metabolic types in the two groups of Han and Uygur patients

As detailed in Table 4, both groups had a higher proportion of patients with a fast metabolism type and a lower proportion with a slow metabolism type. There was no significant difference in metabolic type between the two groups (*χ* ^2^ = 1.906, *P* > 0.05).

**Table 4:** Comparison of metabolic types between the two groups

Metabolic type	Han ethnic group (%)	Uygur ethnic group (%)	Total (%)
Fast metabolic type (CYP2D6*4)	90 (72.0)	93 (76.9)	183 (74.4)
Intermediate metabolic type (CYP2D6*10/*10)	30 (24.0)	21 (17.4)	51 (20.7)
Slow metabolic type (CYP2C19*2/*2, *2/*3 and *3/*3)	5 (4.0)	7 (5.7)	12 (4.9)
Note: *χ* ^2^ = 1.906, *P* = 0.386			

## 5 Discussion

The results of the present study indicate that no CYP2D6*5 allele was identified in the 125 Han patients and 121 Uygur patients with positive premenopausal hormone receptors. This indicates that, to a certain extent, the frequency of this base is low in the Han and Uygur populations of Xinjiang. However, whether its deletion will lead to changes in the metabolic function of the entire *CYP2D6* gene requires clarification via joint research in multiple regions. According to previous reports, the inactive allele, CYP2D6*4, is responsible for 70–90% of the slow metabolic phenotype, with a high incidence of 20–25% in the white population and only <1% in the Asian population [16]. Studies have revealed differences in the frequency of slow and intermediate metabolic alleles among different ethnic groups. Two types of poor metabolisers, those carrying two slow-metabolising alleles, are found in 6–10% of European populations, which is higher than the incidence in East Asian populations (<1%) [17].

However, the frequency of the intermediate metabolic allele, CYP2D6*10, among the East Asian population is significantly higher than among the European population (0.38 in Japan, 0.45 in South Korea, 0.56 in China and <0.02 in Europe) [18]. Moreover, the incidence of poor metabolisers is not high. According to reports, white Europeans (e.g., in Switzerland and Spain) have the highest incidence rate (7%), whereas African American (1.8%) and Chinese (1%) populations have the lowest. In addition, the literature reveals that the incidence of the CYP2D6*10 allele is 40.8% in the Japanese population and 5% in the white population [19]. In Chinese people, CYP2D6*10 is the most commonly mutated allele of *CYP2D6* [20]. The results of this study suggest that *CYP2D6* and *CYP2C19* genotypes need to be tested in patients with breast cancer to allow physicians to tailor the treatment according to these genotypes, thereby reducing mortality rates and personalising the treatment of patients with breast cancer.

In the present study, the allele frequencies of CYP2D6*10 in the Han and Uygur patients were 0.568 and 0.455, respectively (*P* = 0.012), which is consistent with results reported both at home and abroad. The mutation of this gene has led to a decrease in enzyme activity. Among the Xinjiang Uygur patients with hormone-receptor-positive breast cancer, the frequency of the *CYP2D6* intermediate metaboliser (*10/*10) was 16%, which was significantly lower than the frequency of 33% in the Han patients (*P* < 0.05).

The polymorphisms of *CYP2C19* are different in China and other regions in Asia [18]. The two major mutations of *CYP2C19* in the Chinese population are CYP2C19*2 and CYP2C19*3. Extensive metabolisers (*1/*1) and poor metabolisers (*2/*2, *2/*3 and *3/*3) are two phenotypes of *CYP2C19* polymorphisms in this population. In Xinjiang, the *CYP2C19* genotypes of the Han and Uygur populations have significant differences in ethnic distribution. Among the Uygur population, the *CYP2C19* genotypes are significantly more metabolised (CYP2C19*1/*1) than in the Han population. Poor metabolisers (CYP2C19*2/*2, *2/*3 and *3/*3) are also significantly less widespread in the Uygur population than in the Han population [21].

The present study demonstrated that the allele frequencies of CYP2C19*2 in the Han and Uygur patients were 0.38 and 0.488, respectively, whereas the allele frequencies of CYP2C19*3 were 0.068 and 0.041, respectively, and neither CYP2C19*2 nor CYP2C19*3 exhibited racial differences. Bae et al. revealed that the genetic polymorphisms of *CYP2D6* significantly affect metoclopramide pharmacokinetics [22]. In the two groups, more patients (74.4%) belonged to the fast metabolism type. Among these patients, extensive, intermediate and poor metabolisers accounted for 68.2, 17.1 and 7.3%, respectively, and there was no significant racial difference between the two groups of metabolisers. Treatment with tamoxifen was ineffective for the patients with poor metabolisers; in such cases, it may be reasonable to increase the drug dose or perform ovarian castration [23].

This study has a number of limitations. First, the size of the Uygur group was small, meaning the conclusions, especially those for the Uygur population, need to be verified using a larger sample size. Additionally, the conclusions should be interpreted with caution since the population was exclusively from Xinjiang, and generalisability to other populations was not established. Finally, the relationship between patients’ *CYP2D6* and *CYP2C19* genotypes, metabolic types and the long-term efficacy of tamoxifen requires further investigation to provide important information for personalised treatment, improve the safety and efficacy of treatment with tamoxifen and increase the survival rate of patients on a theoretical basis [24]. In future follow-up experiments, we will further expand the research scope and continue to verify the conclusions of this article.

Recent research has shed light on the complex patterns of germline variant frequency in the Chinese population [25]. Notably, significant variation has been observed both between different ethnic groups and within individual ethnic groups. These findings highlight the importance of considering population-specific genetic factors in precision medicine [26].

The present study found no significant racial differences in terms of CYP2D6*5, CYP2C19*1, CYP2C19*3 and tamoxifen metabolism types among Han and Uygur premenopausal patients with breast cancer in Xinjiang. The frequency of the CYP2D6*10 allele was low in the Uygur patients. However, further research on the relationship between this gene and its metabolic type is needed to guide the rational clinical use of tamoxifen.
